# Preclinical evaluation of a SARS-CoV-2 mRNA vaccine PTX-COVID19-B

**DOI:** 10.1126/sciadv.abj9815

**Published:** 2022-01-19

**Authors:** Jun Liu, Patrick Budylowski, Reuben Samson, Bryan D. Griffin, Giorgi Babuadze, Bhavisha Rathod, Karen Colwill, Jumai A. Abioye, Jordan A. Schwartz, Ryan Law, Lily Yip, Sang Kyun Ahn, Serena Chau, Maedeh Naghibosadat, Yuko Arita, Queenie Hu, Feng Yun Yue, Arinjay Banerjee, W. Rod Hardy, Karen Mossman, Samira Mubareka, Robert A. Kozak, Michael S. Pollanen, Natalia Martin Orozco, Anne-Claude Gingras, Eric G. Marcusson, Mario A. Ostrowski

**Affiliations:** 1 Department of Medicine, University of Toronto, Toronto, ON, Canada.; 2 Institute of Medical Science, University of Toronto, Toronto, ON, Canada.; 3 Department of Molecular Genetics, University of Toronto, Toronto, ON, Canada.; 4 Lunenfeld-Tanenbaum Research Institute at Mount Sinai Hospital, Sinai Health System, Toronto, ON, Canada.; 5 Sunnybrook Research Institute, Toronto, ON, Canada.; 6 Providence Therapeutics Holdings Inc., Calgary, AB, Canada.; 7 Department of Immunology, University of Toronto, Toronto, ON, Canada.; 8 Vaccine and Infectious Disease Organization, University of Saskatchewan, Saskatoon, SK, Canada.; 9 Department of Veterinary Microbiology, University of Saskatchewan, Saskatoon, SK, Canada.; 10 Department of Biology, University of Waterloo, Waterloo, ON, Canada.; 11 Department of Medicine, McMaster University, Hamilton, ON, Canada.; 12 Department of Laboratory Medicine and Pathology, University of Toronto, Toronto, ON, Canada.; 13 Marcusson Consulting, San Francisco, CA, USA.; 14 Keenan Research Centre for Biomedical Science of St. Michael’s Hospital, Unity Health Toronto, Toronto, ON, Canada.

## Abstract

Safe and effective vaccines are needed to end the COVID-19 pandemic. Here, we report the preclinical development of a lipid nanoparticle–formulated SARS-CoV-2 mRNA vaccine, PTX-COVID19-B. PTX-COVID19-B was chosen among three candidates after the initial mouse vaccination results showed that it elicited the strongest neutralizing antibody response against SARS-CoV-2. Further tests in mice and hamsters indicated that PTX-COVID19-B induced robust humoral and cellular immune responses and completely protected the vaccinated animals from SARS-CoV-2 infection in the lung. Studies in hamsters also showed that PTX-COVID19-B protected the upper respiratory tract from SARS-CoV-2 infection. Mouse immune sera elicited by PTX-COVID19-B vaccination were able to neutralize SARS-CoV-2 variants of concern, including the Alpha, Beta, Gamma, and Delta lineages. No adverse effects were induced by PTX-COVID19-B in either mice or hamsters. Based on these results, PTX-COVID19-B was authorized by Health Canada to enter clinical trials in December 2020 with a phase 2 clinical trial ongoing.

## Teaser

PTX-COVID19-B is highly immunogenic, safe, and effective in preventing SARS-CoV-2 infection in mice and hamsters.

## INTRODUCTION

COVID-19 (coronavirus disease 2019) caused by SARS-CoV-2 (severe acute respiratory syndrome coronavirus 2) is one of the most severe health crises in human history. Since it was first reported in December 2019, more than 230 million COVID-19 cases and 4.7 million deaths (by 24 September 2021) have been documented, and the pandemic is still spreading (*1*). Public health measures, such as social distancing, mask wearing, contact tracing, quarantine, and national lockdowns, have only partially stymied the pandemic. Some treatment regimens were shown to suppress SARS-CoV-2 replication and/or reduce the number of severe COVID-19 cases and deaths (*2*, *3*). Despite these advances in prevention and treatment, safe and effective SARS-CoV-2 vaccines are ultimately needed for sustainable control of the pandemic and a return to normalcy.

With unprecedented speed, hundreds of SARS-CoV-2 vaccine candidates have been designed and produced, with more than 300 tested in animals since the beginning of the pandemic (*4*, *5*). Among them, 21 have been approved for emergency use in humans, and dozens, including the PTX-COVID19-B reported here, are at various stages of clinical trials (*4*). Given the current world population requiring vaccination, the variable conditions of public health infrastructure in different countries, and the rapid emergence of COVID-19 variants of concern (VOCs) that may escape vaccine-induced immune responses (*6*–*9*), continued and concerted global efforts in SARS-CoV-2 vaccine research, development, manufacturing, and deployment are required to end the COVID-19 pandemic (*10*).

SARS-CoV-2 is an enveloped positive-sense RNA virus that uses the spike protein (S) on its surface to bind the angiotensin-converting enzyme 2 (ACE2) on host cells for entry to initiate replication (*11*–*15*). The S protein has two subunits: S1 and S2. S1 is responsible for binding to ACE2 through its receptor binding domain (RBD). Once bound, S1 is shed from the envelope, exposing S2, which is then inserted into the host cell membrane to mediate fusion of virus envelope and cell membrane to release the viral genetic material into the host cells for replication. In contrast to SARS-CoV and other group 2B coronaviruses, SARS-CoV-2 has a furin cleavage site between the S1 and S2 subunits, which promotes infection of cells expressing the transmembrane serine protease 2 (TMPRSS2) on their surface, e.g., human respiratory tract epithelial cells (*11*, *12*). The S protein is also the main target of host-generated neutralizing antibodies (nAbs) that can inhibit SARS-CoV-2 infection, e.g., by blocking its binding to ACE2 (*16*, *17*). Thus, most of the current SARS-CoV-2 vaccines use S protein as the immunogen.

mRNA-based vaccines are attractive platforms for prophylactic SARS-CoV-2 vaccine candidates because of their unique advantages, including rapid large-scale production, strong immunogenicity in both humoral and cellular immunity, and ease of adaptation to tackle the emerging VOCs (*18*, *19*). Two SARS-CoV-2 mRNA vaccines were the earliest to enter phase 3 clinical trials, showing both high efficacy and safety, and were the first to be approved for emergency use in humans (*20*, *21*). Here, we report the preclinical results of another SARS-CoV-2 mRNA vaccine, PTX-COVID19-B. Distinct from the approved mRNA vaccines, PTX-COVID19-B uses native S protein sequence (without the 2P, K986P and V987P, mutation) from SARS-CoV-2 Wuhan-Hu-1 isolate (GenBank accession number MN908947.3) but replaces D614 with G614. We found that PTX-COVID19-B elicited potent humoral and cellular immune responses in mice and protected both mice and hamsters from SARS-CoV-2 challenges. On the basis of these results, PTX-COVID19-B was authorized by Health Canada to enter clinical trials with a phase 2 clinical trial underway.

## RESULTS

### SARS-CoV-2 mRNA vaccine candidates

**Fig. 1.**
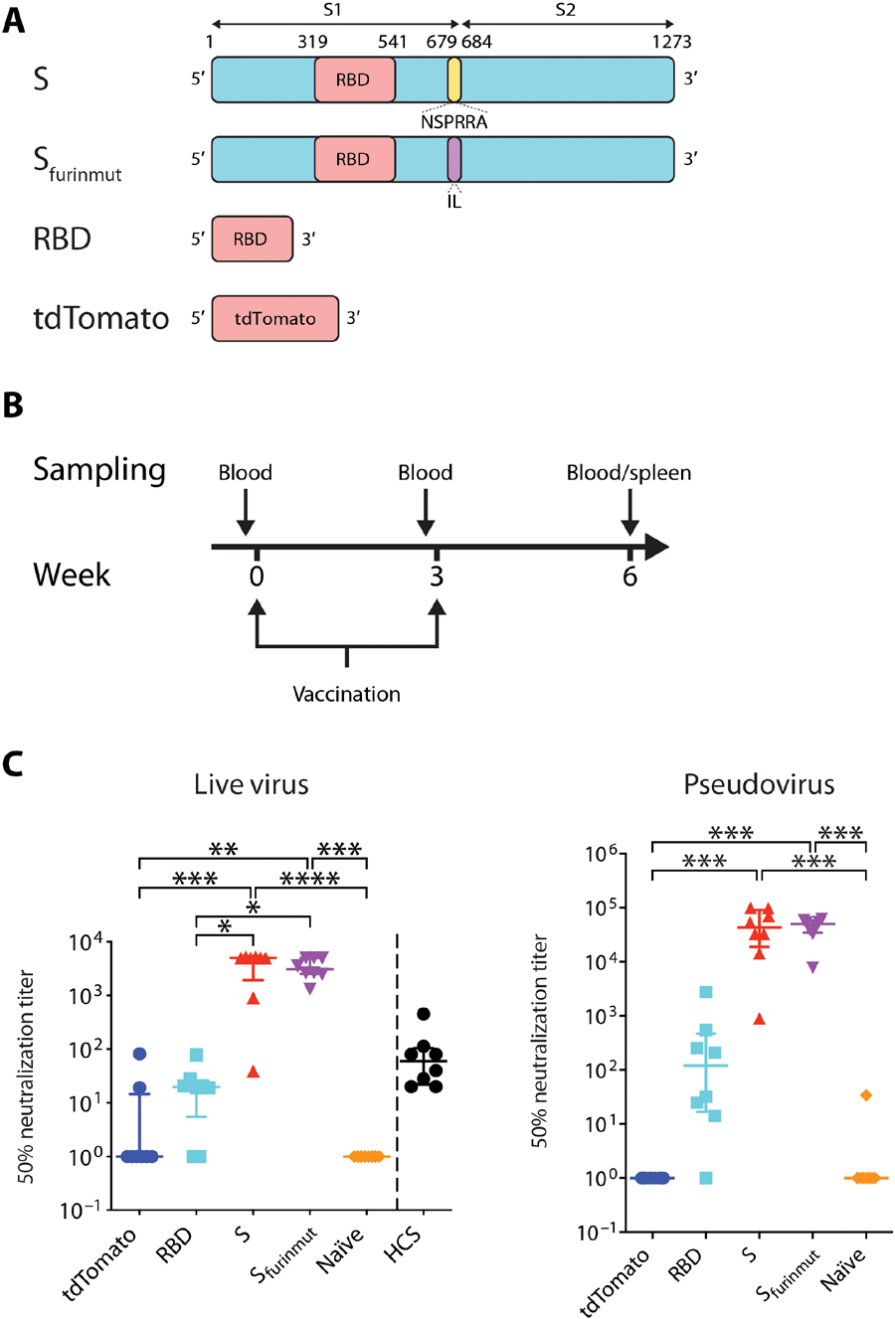
SARS-CoV-2 mRNA vaccine candidates elicit SARS-CoV-2 nAbs in mice. (**A**) Schematic representation of the mRNA vaccine constructs. S, SARS-CoV-2 Spike mRNA (amino acids 1 to 1273); S_furinmut_, SARS-CoV-2 Spike mRNA (amino acids 1 to 1273) in which the furin cleavage site was removed by replacing NSPRRA (amino acids 679 to 684) with IL; RBD, SARS-CoV-2 RBD (receptor binding domain) mRNA (amino acids 319 to 541); tdTomato, control mRNA encoding tdTomato; S1, S1 subunit of SARS-CoV-2 Spike (amino acids 1 to 685); S2, S2 subunit of SARS-CoV-2 Spike (amino acids 686 to 1273). (**B**) Mice vaccination regimen. Six- to 8-week-old mice were vaccinated twice with a 3-week interval. One day before each vaccination, peripheral blood was collected from the mice. Three weeks after the second vaccination, mice were humanely euthanized, and blood and spleens were collected from the mice. (**C**) C57BL/6 mice (*n* = 8 per group) were vaccinated with 20 μg of mRNA vaccine candidates (S, S_furinmut_, and RBD) or control mRNA tdTomato or DPBS for naïve control mice. Three weeks after the second vaccination, blood was collected to test neutralization of SARS-CoV-2 authentic virus or pseudovirus by the sera. For comparison, convalescent sera from eight SARS-CoV-2–infected human subjects (HCS in the graph) were also tested for neutralization of SARS-CoV-2 authentic virus. Each symbol represents one mouse or person. Samples that did not neutralize viruses at the lowest dilution (1:20 for real virus and 1:40 for pseudovirus) are designated an ID_50_ titer of 1. For each group, the long horizontal line indicates the median, and the short lines below and above the median indicate the 25th and 75th percentiles. *P < 0.05, **P < 0.01, ***P < 0.001, and ****P < 0.0001 as determined by one-way ANOVA (Kruskal-Wallis test) followed by Dunn’s multiple comparison test.

We first designed three SARS-CoV-2 mRNA vaccine candidates and compared their immunogenicity in mice: an RBD construct (amino acids 319 to 541), a full-length S construct (amino acids 1 to 1273), and an S_furinmut_ construct in which NSPRRA (amino acids 679 to 684) in the full-length S were replaced with IL to remove the furin cleavage site between S1 and S2 (Fig. 1A). The coding sequences of all three constructs were based on the S protein from the SARS-CoV-2 Wuhan-Hu-1 isolate (GenBank accession number MN908947.3) except for a D614G substitution in the S and S_furinmut_ constructs to match this amino acid location to that of the current dominant circulating strains (*22*, *23*). Codons and sequences in these mRNA constructs were optimized by using Providence’s proprietary algorithm to increase the translation of the encoded proteins. The RBD construct was included for testing since it is the main target of nAbs (*16*, *17*). The S_furinmut_ construct was made, as it has been shown that removing the furin cleavage sites in some viral envelope proteins can enhance their expression and stability, especially when their ectodomains are expressed as soluble proteins (*24*–*27*). Expression of the protein encoded by these mRNA constructs on the surface of transfected human embryonic kidney (HEK) 293T cells was detected by flow cytometry, and in the supernatant by enzyme-linked immunosorbent assay (ELISA), using the RBD-specific neutralizing monoclonal antibody (mAb) COV2-2165 (fig. S1, A and B) (*17*). As expected, both the S and S_furinmut_ proteins encoded by the mRNA constructs were expressed on the cell surface. The expressed RBD construct was detected only in the supernatant, consistent with its expression in soluble form. Some S protein could also be detected in the supernatant of the S mRNA–transfected cells, possibly due to the furin cleavage of the membrane-bound S protein.

To compare the immunogenicity of the three mRNA constructs, female C57BL/6 mice were vaccinated twice, 3 weeks apart, with 20 μg of each of the constructs formulated in lipid nanoparticle (LNP) (Fig. 1B). Control mice received either 20 μg of an mRNA encoding tdTomato in the same LNP or the same volume of Dulbecco’s phosphate-buffered saline (DPBS). Blood was collected 3 weeks after boost vaccination, and the presence of nAbs in the sera was measured by a microneutralization assay using a SARS-CoV-2 virus isolated from a SARS-CoV-2 patient [SARS-CoV-2-SB2-P3 PB clone 1 (*28*)] (Fig. 1C). We found that the full-length S mRNA candidate elicited the highest nAb levels in the sera of vaccinated mice. The level of nAb induced by the S_furinmut_ mRNA candidate was lower than that elicited by the full-length S mRNA candidate, but the difference was not statistically significant. Median nAb 50% neutralization (ID_50_) titer was 4991 [interquartile range (IQR), 1927 to 5188] and 3085 (IQR, 2528 to 4991) for the S and S_furinmut_ mRNA, respectively. The RBD mRNA candidate induced low nAb levels (median, 19.8; IQR, 5.5 to 26.0). No nAb was detected in the sera from control mice receiving DPBS, and low levels of nAb were detected in the sera of two of eight control mice receiving the tdTomato mRNA (nAb ID_50_ titer was 19 and 82, respectively). Notably, the median serum nAb titer of the S and S_furinmut_ mRNA–vaccinated mice was 83.1-fold and 51.4-fold higher than that of eight COVID-19 convalescent patients (median nAb ID_50_ titer, 60; IQR, 22.0 to 104.8; two patients in each category of severe, moderate, mild, and asymptomatic SARS-CoV-2 infections), respectively (Fig. 1C). Similar results were obtained when the serum nAb was measured by a lentivirus-based SARS-CoV-2 pseudovirus neutralization assay, although the nominal nAb ID_50_ titers from the pseudovirus assay were usually higher than those from the microneutralization assay (Fig. 1C). On the basis of these results, the full-length S mRNA construct, hereafter named PTX-COVID19-B, was chosen for further testing and moved into the next stages of development.

### Humoral immune responses elicited by PTX-COVID19-B vaccination

**Fig. 2.**
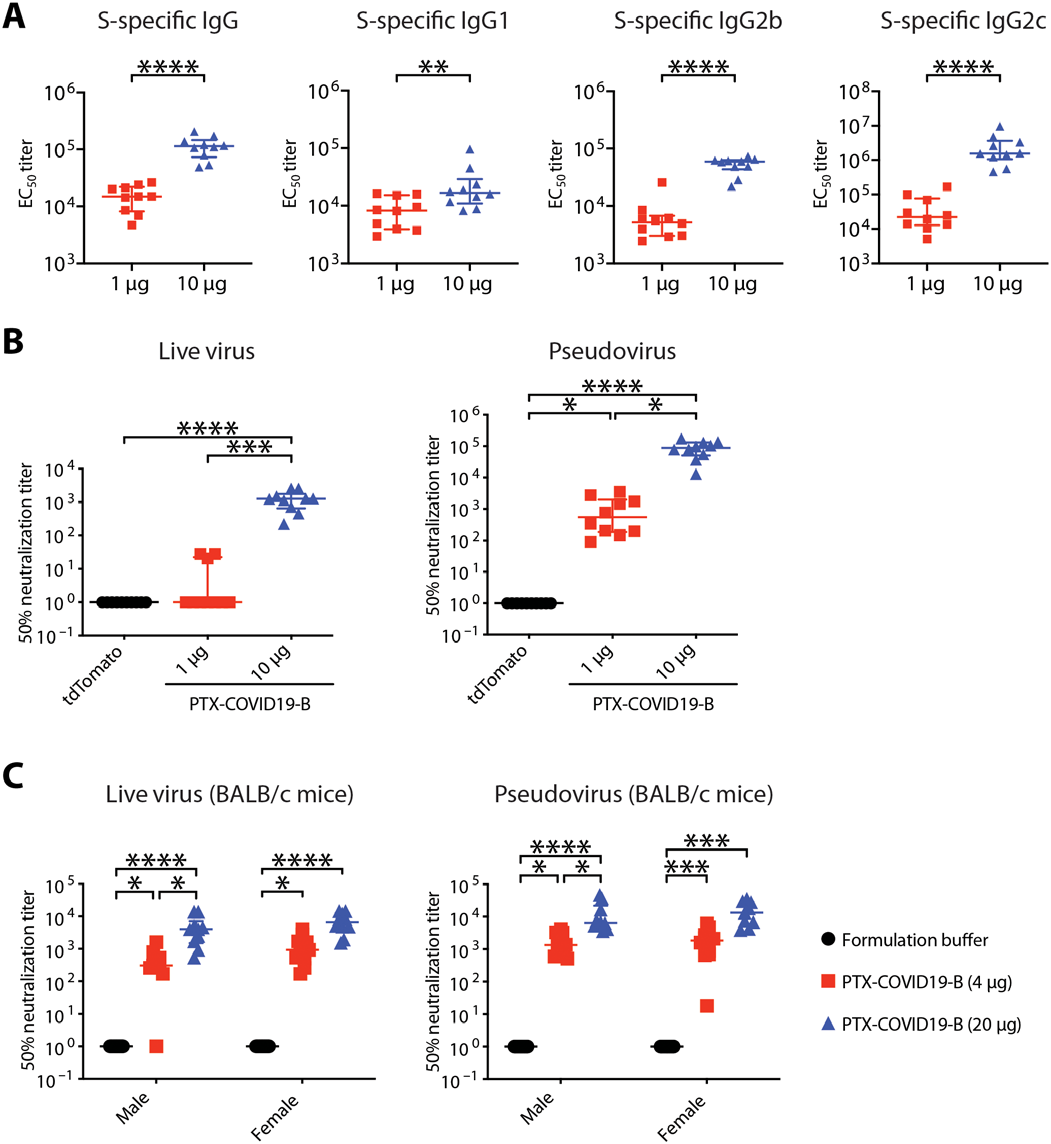
PTX-COVID19-B elicits potent humoral immune responses in mice. (**A** and **B**) Female C57BL/6 mice were vaccinated with 1 or 10 μg of PTX-COVID19-B or 10 μg of control tdTomato mRNA. Three weeks after the second vaccination, blood was collected to detect (A) S-specific binding antibodies in the mouse sera as measured by ELISA and (B) neutralization of SARS-CoV-2 authentic virus or pseudovirus by the mouse sera. Shown in (A) are EC_50_ titers. *N* = 10 for each of the PTX-COVID19-B group. Shown in (B) are ID_50_ titers. *N* = 10 per group. (**C**) Six- to 8-week-old male and female BALB/c mice were vaccinated with 4- or 20-μg doses of PTX-COVID19-B or formulation buffer as a control. Three weeks after the second vaccination, blood was collected to detect serum neutralization of SARS-CoV-2 authentic virus or pseudovirus by the mouse sera. Shown are ID_50_ titers. *N* = 10 per group except for *n* = 9 for the female 20 μg–dosed PTX-COVID19-B group in the pseudovirus assay. In (B) and (C), samples that did not neutralize viruses at the lowest dilution (1:20 for real virus and 1:40 for pseudovirus) are designated an ID_50_ titer of 1. Each symbol represents one mouse. For each group, the long horizontal line indicates the median, and the short lines below and above the median indicate the 25th and 75th percentiles. *P < 0.05, **P < 0.01, ***P < 0.001, and ****P < 0.0001 as determined by one-way ANOVA (Kruskal-Wallis test) followed by Dunn’s multiple comparison test.

To further evaluate the immunogenicity of PTX-COVID19-B, female C57BL/6 mice were vaccinated twice, 3 weeks apart, with 1- or 10-μg doses of PTX-COVID19-B or, as control, 10 μg of LNP-formulated tdTomato mRNA. Three weeks after the boost vaccination, blood and spleens were collected from the mice to measure humoral and cellular immune responses. We first used an ELISA assay to measure S-specific binding antibodies in the sera of the mice. As shown in Fig. 2A, both 1- and 10-μg doses of PTX-COVID19-B elicited very strong S-specific total immunoglobulin G (IgG), IgG1, IgG2b, and IgG2c responses [median half maximal effective concentration (EC_50_) titers for 1 and 10 μg of PTX-COVID19-B are, respectively, as follows: 1.5 × 10^4^ (IQR, 8.1 × 10^3^ to 2.2 × 10^4^), 1.1 × 10^5^ (IQR, 7.3 × 10^4^ to 1.5 × 10^5^) for total IgG; 8.3 × 10^3^ (IQR, 3.9 × 10^3^ to 1.5 × 10^4^), 1.7 × 10^4^ (IQR, 1.1 × 10^4^ to 2.9 × 10^4^) for IgG1; 5.2 × 10^3^ (IQR, 3.0 × 10^3^ to 6.7 × 10^3^), 5.9 × 10^4^ (IQR, 4.4 × 10^4^ to 6.3 × 10^4^) for IgG2b; 2.2 × 10^4^ (IQR, 1.3 × 10^4^ to 7.6 × 10^4^), 1.6 × 10^6^ (IQR, 1.1 × 10^6^ to 3.6 × 10^6^) for IgG2c]. The 10-μg dose of PTX-COVID19-B usually induced higher S-specific binding antibodies than the 1-μg dose. The preponderance of the T helper 1 (T_H_1) antibody (IgG2b and IgG2c) over the T_H_2 antibody (IgG1) also indicated that PTX-COVID19-B induced a T_H_1-biased antibody response. Very low levels of anti-S antibodies were detected in the sera of control mice receiving the tdTomato mRNA.

We then measured nAb against SARS-CoV-2 in these C57BL/6 mouse sera. Results of SARS-CoV-2 authentic virus microneutralization assay showed that the 10-μg dose of PTX-COVID19-B elicited high nAb levels (median nAb ID_50_ titer was 1259; IQR, 652.7 to 1770), which was 21.0-fold higher than that of the eight COVID-19 convalescent patients (Figs. 1C and 2B). Low levels of nAb were induced by the 1-μg dose of PTX-COVID19-B, which, for most mice, could only be detected by the pseudovirus assay, which is more sensitive (Fig. 2B). nAb was not detected in the sera of the mice receiving tdTomato mRNA by either assay.

To further verify the ability of PTX-COVID19-B in inducing a nAb response against SARS-CoV-2 virus, we vaccinated a different strain of mice, BALB/c, and included both sexes in the vaccination, using the same vaccination regimen as described above. Three weeks after the boost vaccination, sera were collected and nAb levels were measured. As shown in Fig. 2C, both 4- and 20-μg doses of PTX-COVID19-B elicited potent nAb responses in both male and female BALB/c mice. The 20-μg dose of PTX-COVID19-B induced higher nAb titers than 4 μg, although this only reached statistical significance in male mice. nAb was not detected in the sera of the control mice receiving formulation buffer.

### Neutralization of VOCs by PTX-COVID19-B elicited immune sera

**Fig. 3.**
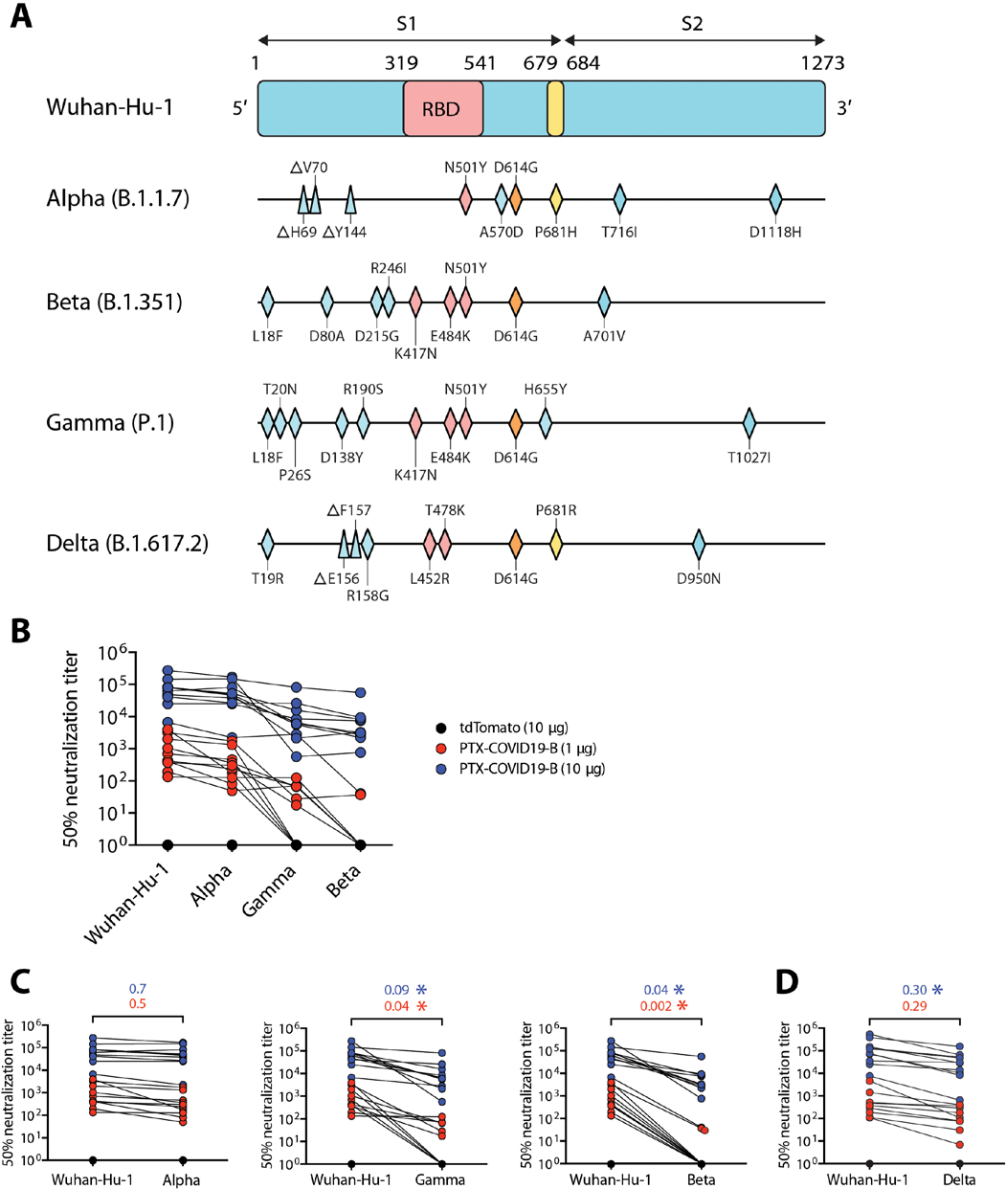
Neutralization of VOCs by immune sera from PTX-COVID19-B vaccinated mice. (**A**) Schematic representation of S proteins from SARS-CoV-2 VOCs, highlighting mutated amino acids compared to the ancestral Wuhan-Hu-1 isolate (triangles denoting deletions and rhombuses denoting replacements). (**B** to **D**) Neutralization of pseudoviruses bearing S protein from VOCs and Wuhan-Hu-1 isolate. C57BL/6 mice immune sera shown in Fig. 2 (A and B) were used in the neutralization. Shown in (B) are ID_50_ titers across all pseudoviruses except VOC Delta. Shown in (C) are pairwise comparisons of the ID_50_ titers between VOCs and the Wuhan-Hu-1 isolate. Shown in (D) is a pairwise comparison of the ID_50_ titers between VOC Delta and the Wuhan-Hu-1 isolate. The numbers above the brackets in (C) and (D) are the ratios of the median ID_50_ titers against the VOCs to the titers against Wuhan-Hu-1 isolate (blue, 10-μg PTX-COVID19-B group; red, 1-μg PTX-COVID19-B group). Each symbol represents one mouse. *P < 0.05 as determined by two-tailed paired *t* test.

VOCs evade neutralization by sera from SARS-CoV-2 vaccinees, raising concerns about the efficacy of current SARS-CoV-2 vaccines. Using the pseudovirus assay (here, lentivirus particles pseudotyped to harbor the same mutations in the S protein that are found in circulating VOCs), we measured neutralization of VOCs by immune sera from PTX-COVID19-B–vaccinated C57BL/6 mice. These VOCs include the Alpha (B.1.1.7) lineage first detected in the United Kingdom (*29*), the Beta (B.1.351) lineage in South Africa (*30*), the Gamma (P.1) lineage in Brazil (*31*), and the Delta (B.1.617.2) lineage in India (*32*) (Fig. 3A). As shown in Fig. 3 (B and C), compared to the Wuhan-Hu-1–pseudotyped lentivirus, Alpha pseudovirus was slightly resistant to neutralization by the mouse immune sera, but this difference did not reach statistical significance. However, Beta and Gamma pseudoviruses significantly reduced the neutralizing potency of the immune sera. For example, for the mice receiving a 10-μg dose of PTX-COVID19-B, median serum nAb ID_50_ titer against Beta and Gamma pseudoviruses was decreased to 4 and 9%, respectively, compared to Wuhan-Hu-1 pseudovirus. Pseudovirus bearing S protein from VOC Delta, the current predominant circulating SARS-CoV-2 lineage around the world, was more resistant to neutralization by the mouse immune sera than Alpha pseudovirus but much more sensitive to the neutralization than Beta and Gamma pseudoviruses (Fig. 3D). Note that the nominal serum nAb ID_50_ titers of the immune sera from the 10-μg PTX-COVID19-B–vaccinated mice against these VOCs are still very high, with median titers ranging from 3.2 × 10^3^ to 6.4 × 10^4^ for the different VOCs tested.

### Cellular immune responses elicited by PTX-COVID19-B vaccination

**Fig. 4.**
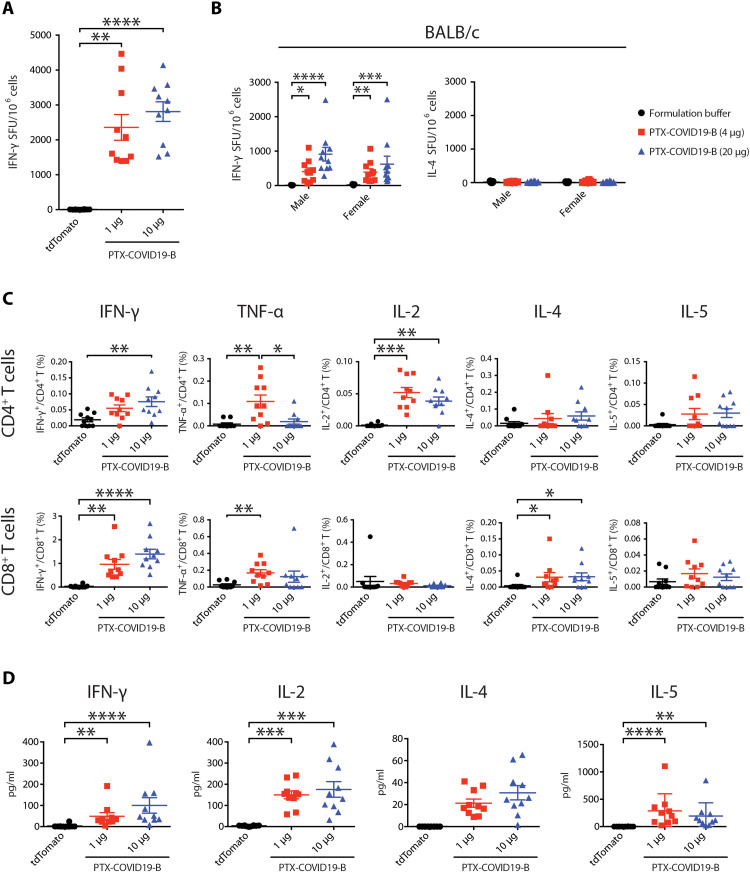
PTX-COVID19-B elicits robust cellular immune responses in mice. (**A**, **C**, and **D**) Female C57BL/6 mice were vaccinated with 1- or 10-μg doses of PTX-COVID19-B or 10 μg of tdTomato mRNA. (**B**) Male and female BALB/C mice were vaccinated with 4- or 20-μg doses of PTX-COVID19-B or formulation buffer as a control. Three weeks after the second vaccination, spleens were collected and the splenocytes were stimulated with SARS-CoV-2 S peptide pool to detect cytokine production, as measured by ELISPOT shown in (A) and (B), intracellular cytokine staining and flow cytometry shown in (C), and a multiplex immunoassay shown in (D). Shown in (A) are the numbers of IFN-γ spot-forming units (SFU) per million splenocytes (*n* = 10 per group), (B) the numbers of IFN-γ and IL-4 spot-forming units per million splenocytes (*n* = 9 for each of the formulation groups and *n* = 10 for each of the PTX-COVID19-B groups), (C) percentage of cytokine-producing cells in CD4^+^ or CD8^+^ T cells (*n* = 10 per group), and (D) quantity of the cytokines in the supernatants of the stimulated splenocytes (*n* = 10 per group). Each symbol represents one mouse. For each group, the long horizontal line indicates the mean, and the short lines below and above the mean indicate the SEM. *P < 0.05, **P < 0.01, ***P < 0.001, and ****P < 0.0001 as determined by one-way ANOVA (Kruskal-Wallis test) followed by Dunn’s multiple comparison test.

C57BL/6 mice vaccinated with 1 and 10 μg of PTX-COVID19-B were humanely euthanized 21 days after the boost vaccination, and splenocytes were stimulated with an S peptide pool (315 15-mer peptides with 11–amino acid overlaps encompassing the entire S protein) to measure interferon-γ (IFN-γ)–producing cells by enzyme-linked immunospot assay (ELISPOT) (Fig. 4A). The 1- and 10-μg PTX-COVID19-B–vaccinated mice had 2356 ± 369.7 and 2810 ± 280.9 (means ± SEM) IFN-γ spot-forming units per million splenocytes, respectively, suggesting a strong T_H_1 response. Moreover, when splenocytes from both sexes of BALB/c mice immunized with 4 and 20 μg of PTX-COVID19-B were evaluated via IFN-γ and interleukin-4 (IL-4) ELISPOT, several hundreds of IFN-γ spot-forming units per million splenocytes on average were detected in immunized mice, while very few IL-4 spot-forming units above the background were detected (Fig. 4B). This indicates a strong T_H_1 response driven by the vaccination even in a mouse strain (BALB/c) with a tendency for T_H_2 responses.

In addition, cytokine-producing CD4^+^ and CD8^+^ T cells in splenocytes of C57BL/6 mice immunized with 1 and 10 μg of vaccine were analyzed by flow cytometry following overnight S peptide pool stimulation and intracellular cytokine staining (Fig. 4C). CD4^+^ T cells had increased percentages of IFN-γ–, tumor necrosis factor–α (TNF-α)–, and IL-2–producing cells and very low percentages of IL-4– and IL-5–producing cells, indicating a strong induction of a T_H_1 response. CD8^+^ T cells showed a high number of IFN-γ–producing cells, which was higher in percentage than that of CD4^+^ T cells (Fig. 4C and fig. S2). These results contrast with the T cell responses identified in patients with COVID-19, where CD4^+^ T cell responses against SARS-CoV-2 outweigh CD8^+^ T cells (*33*–*37*).

Furthermore, cytokines were measured in the supernatants of S peptide pool–stimulated splenocytes from C57BL/6 vaccinated mice by a multiplex immunoassay (Fig. 4D), and the results confirmed a strong production of IFN-γ and IL-2 that correlates with the flow cytometry and ELISPOT data. Collectively, these results indicate that PTX-COVID19-B vaccination induced robust T_H_1-biased CD4^+^ and CD8^+^ T cell responses.

### PTX-COVID19-B protecting mice from SARS-CoV-2 challenge

**Fig. 5.**
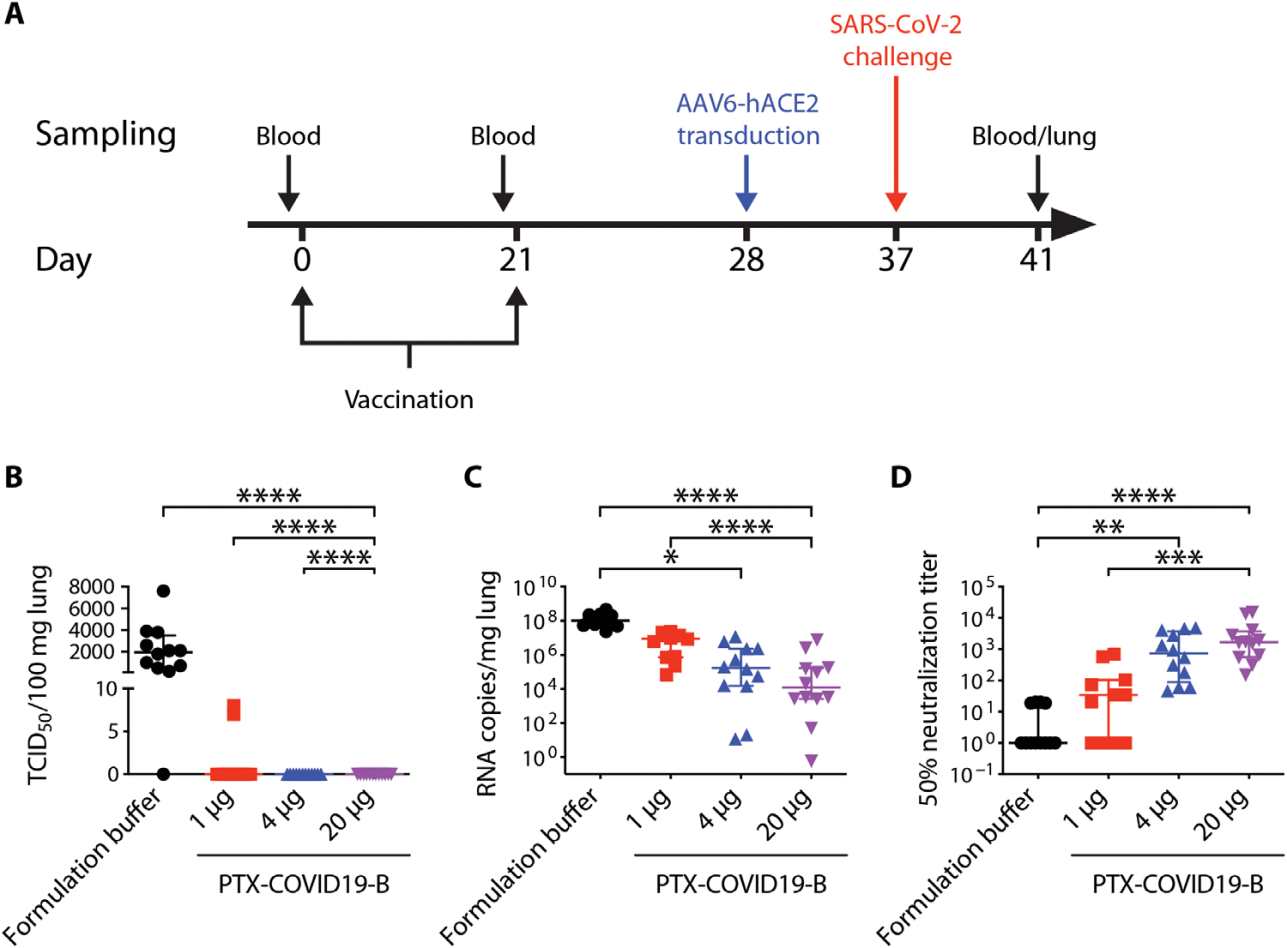
PTX-COVID19-B protects mice from SARS-CoV-2 challenge. (**A**) Mouse vaccination and challenge regimen. Six- to 8-week-old female C57BL/6 mice (*n* = 10 per group) were vaccinated twice with 1-, 4-, or 20-μg doses of PTX-COVID19-B or formulation buffer as a control. One week after the second vaccination, mice were intranasally transduced with AAV6-hACE2. Nine days after the transduction, mice were intranasally challenged with SARS-CoV-2. One day before each vaccination, blood was collected from the mice. Four days after SARS-CoV-2 challenge, mice were humanely euthanized, and blood and lungs were collected from the mice. (**B**) Amount of infectious SARS-CoV-2 virus and (**C**) SARS-CoV-2 RNA in the lungs of the mice. Shown in (B) is TCID_50_/100 mg lung tissue (*n* = 10 per group) and (C) RNA copies/mg lung tissue (*n* = 10 per group). (**D**) Neutralization of SARS-CoV-2 authentic virus by the mouse sera collected 4 days after SARS-CoV-2 challenge. Shown are ID_50_ titers (*n* = 10 per group). Samples that did not neutralize viruses at the lowest dilution (1:20) are designated an ID_50_ titer of 1. Each symbol represents one mouse. For each group, the long horizontal line indicates the median, and the short lines below and above the median indicate the 25th and 75th percentiles. *P < 0.05, **P < 0.01, ***P < 0.001, and ****P < 0.0001 as determined by one-way ANOVA (Kruskal-Wallis test) followed by Dunn’s multiple comparison test.

Since wild-type mice are not susceptible to ancestral SARS-CoV-2 infection, we used an AAV6 (adeno-associated virus type 6)–human ACE2 (hACE2) mouse model to test whether PTX-COVID19-B can protect mice from SARS-CoV-2 infection. A similar mouse model using AAV type 9 to transduce hACE2 into mice was reported to support SARS-CoV-2 replication in mouse lungs (*38*). In our model, mice were first transduced with AAV6-hACE2 intranasally to express hACE2 in their respiratory tracts and, 9 days later, were intranasally inoculated with 10^5^ 50-percent tissue culture infectious dose (TCID_50_) SARS-CoV-2 (isolate Canada/ON/VIDO-01/2020, a B lineage isolate with D614) (Fig. 5A and fig. S3). As shown in fig. S3 and consistent with the previous report, AAV6-mediated hACE2 transduction induced susceptibility to SARS-CoV-2 infection as shown by the detection of infectious SARS-CoV-2 in the lungs of AAV6-hACE2 mice but not in control mice transduced with AAV6-luciferase (fig. S3A). Using a real-time reverse transcription polymerase chain reaction (RT-PCR) assay targeting the SARS-CoV-2 envelope (E) gene, we also detected a high amount of SARS-CoV-2 genomic RNA in the lungs from both AAV6-hACE2 and AAV6-luciferase transduced mice, although the genomic RNA copy numbers were much lower in the lungs of the AAV6-luciferase transduced mice than in those of the AAV6-hACE2 mice (fig. S3B).

Having confirmed that the AAV6-hACE2 mouse model was susceptible to SARS-CoV-2 infection, we vaccinated four groups of C57BL/6 mice twice with three different doses of PTX-COVID19-B (1, 4, and 20 μg) and, as a control, the formulation buffer for PTX-COVID19-B (Fig. 5). One week after the boost vaccination, all mice were transduced with AAV6-hACE2 followed by SARS-CoV-2 challenge 9 days later. Four days after challenge [4 days postinfection (dpi)], lungs were collected and infectious SARS-CoV-2 virus in the lung tissue homogenates was quantified. Infectious SARS-CoV-2 virus was present in the lungs from 11 of 12 control mice receiving the formulation buffer (median TCID_50_/100 mg lung was 1950; IQR, 550 to 3500; Fig. 5B). In contrast, no infectious virus was detected in the lungs from mice vaccinated with 4- or 20-μg doses of PTX-COVID19-B. For the mice receiving a 1-μg dose of PTX-COVID19-B, low levels of infectious virus were found in only 2 of 12 mice (TCID_50_/100 mg lung = 7 and 8, respectively). SARS-CoV-2 genomic RNA could be detected in the lungs of all mice using the E gene–specific real-time RT-PCR assay, but was reduced, on average, to 0.6, 1.3, and 6.3% in the mice receiving 20-, 4-, and 1-μg doses of PTX-COVID19-B, respectively, compared to the mice receiving formulation buffer (Fig. 5C). We also measured the nAb titers in the sera collected at 4 dpi and found high levels of nAb in the sera from the mice vaccinated with 20- and 4-μg doses of PTX-COVID19-B and moderate levels of nAb from the mice receiving a 1-μg dose of PTX-COVID19-B (Fig. 5D). Given the short time after SARS-CoV-2 challenge (4 dpi), the nAb levels in these mouse sera were most likely elicited by the vaccination, not induced or boosted by the SARS-CoV-2 infection. Notably, the serum nAb ID_50_ titers negatively correlate with the quantities of the infectious SARS-CoV-2 virus and the genomic RNA in the lungs (fig. S4). Logistic regression modeling of the nAb ID_50_ titers and the virus TCID_50_ values indicates that a threshold nAb ID_50_ titer of 654.9 against authentic virus predicts a 95% probability of protection from productive SARS-CoV-2 infection. Together, these data indicate that PTX-COVID19-B completely protected mice from pulmonary infection by SARS-CoV-2, even at a low dose of 4 μg.

### PTX-COVID19-B protecting hamsters from SARS-CoV-2 challenge

**Fig. 6.**
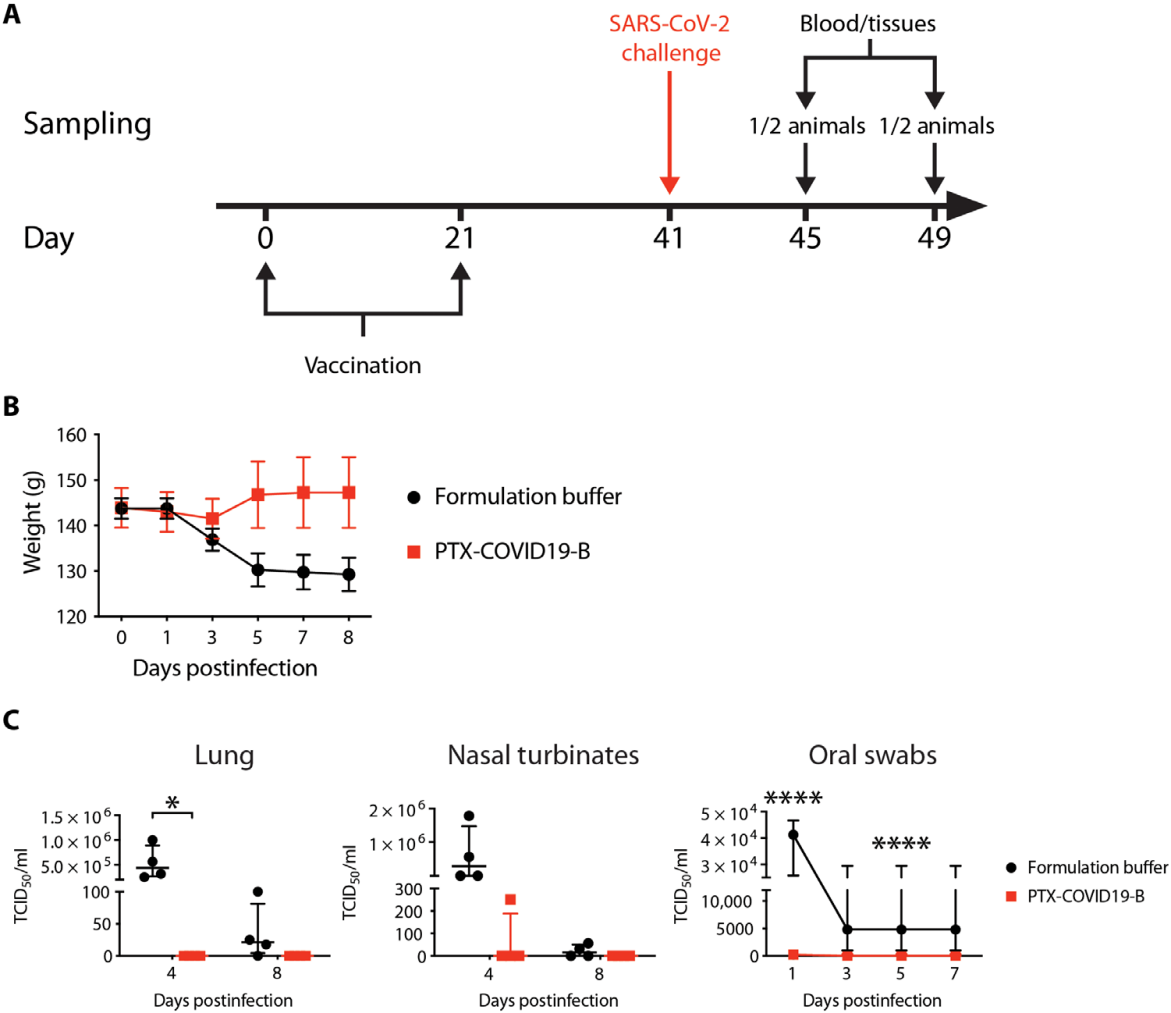
PTX-COVID19-B protects hamsters from SARS-CoV-2 challenge. (**A**) Hamster vaccination and SARS-CoV-2 challenge regimen. Six- to 10-week-old male Syrian hamsters (*n* = 8) were vaccinated with a 20-μg dose of PTX-COVID19-B or formulation buffer twice with a 3-week interval. Twenty days after the second vaccination, hamsters were challenged intranasally with SARS-CoV-2. Four and 8 days after the challenge, half of the animals in each group (four animals per group) were humanely euthanized, and blood and tissues were collected. (**B**) Body weight of the hamsters. Symbols indicate the means, and the error bars indicate the SEM for each group. *N* = 8 per group from days 0 to 3. *N* = 4 from days 5 to 8. (**C**) Amount of infectious SARS-CoV-2 virus in the hamster tissues. Shown are TCID_50_ values per milliliter of the tissue homogenates (lung and nasal turbinates) or per milliliter of oral swab samples. For lungs and nasal turbinates, each symbol represents one hamster (*n* = 4 per group per time point), the long horizontal line indicates the median, and the short lines below and above the median indicate the 25th and 75th percentiles. For oral swabs, symbols indicate the medians, and the error bars indicate the 25th and 75th percentiles (*n* = 8 per group from days 0 to 3 and *n* = 4 from days 5 to 7). *P < 0.05 and ****P < 0.0001 as determined by two-way ANOVA followed by Sidak’s multiple comparison test.

Syrian hamsters are susceptible to and can transmit SARS-CoV-2 infection, mimicking some aspects of SARS-CoV-2 infection in humans (*39*, *40*). We thus tested the efficacy of PTX-COVID19-B in Syrian hamsters (Fig. 6). Two groups of hamsters (*n* = 8) were vaccinated twice with a 3-week interval with either a 20-μg dose of PTX-COVID19-B or the formulation buffer. Twenty days after boost vaccination, all hamsters were challenged intranasally with 10^4^ TCID_50_ SARS-CoV-2 (isolate Canada/ON/VIDO-01/2020, a B lineage isolate with D614). Body weight of the hamsters was measured 1 day before the SARS-CoV-2 challenge and then at 1, 3, 5, 7, and 8 dpi. Oral swabs were taken from the hamsters at 1, 3, 5, and 7 dpi. At 4 and 8 dpi, half (*n* = 4) of the hamsters from each group were humanely euthanized, and nasal turbinates and lungs were collected.

When compared to prechallenge, the body weight of the control hamsters decreased from 3 to 8 dpi, while that of the PTX-COVID19-B–vaccinated hamsters decreased slightly at 3 dpi and then increased from 4 to 8 dpi (Fig. 6B). We then measured the amount of infectious virus in the respiratory tracts of the hamsters (Fig. 6C). No infectious SARS-CoV-2 virus was detected in the lungs of PTX-COVID19-B–vaccinated hamsters at 4 and 8 dpi. In the lungs of control hamsters, a large amount of infectious virus was found at 4 dpi (median TCID_50_ = 4.4 × 10^5^; IQR, 2.7 × 10^5^ to 8.9 × 10^5^), and low levels of infectious SARS-CoV-2 virus (median TCID_50_ = 21.5; IQR, 4.4 to 81.3) were still present in three of four animals at 8 dpi. Infectious virus was also found in the nasal turbinates of control hamsters at 4 and 8 dpi, respectively (4 dpi median TCID_50_ = 2.8 × 10^5^; IQR, 4.4 × 10^3^ to 1.5 × 10^6^; 8 dpi median TCID_50_ = 15.8; IQR, 0 to 50.1). No infectious virus was detected in the nasal turbinates of PTX-COVID19-B–vaccinated hamsters at 4 and 8 dpi, except in one animal, where low level (TCID_50_ = 251) was detected at 4 dpi (Fig. 6C). Similar results were observed in the oral swabs, where little or no infectious SARS-CoV-2 virus was detected in the samples of PTX-COVID19-B–vaccinated hamsters, but high levels of the virus were detected in control hamsters from 1 to 5 dpi (Fig. 6C).

**Fig. 7.**
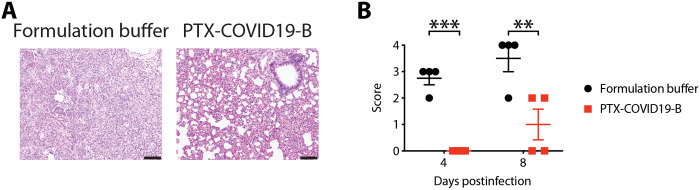
PTX-COVID19-B protects hamster lungs from the pathology caused by SARS-CoV-2 challenge. Lung tissues obtained from the hamsters in Fig. 6 were fixed in formalin and stained with hematoxylin and eosin. (**A**) Representative micrographs of the hamster lungs, showing extensive acute and mixed inflammatory cell infiltrates in bronchiole and alveoli in a control hamster receiving formulation buffer and paucity of inflammation in a vaccinated animal after challenged with SARS-CoV-2. Scale bars, 100 μm. (**B**) Pathology score of the hamster lungs. Shown are semiquantitative pathology scores of the hamster lungs. Each symbol represents one hamster (*n* = 4 per group per time point). The long horizontal lines indicate the means, and the short lines below and above the mean indicate the SEM. **P < 0.01 and ***P < 0.001 as determined by two-way ANOVA followed by Sidak’s multiple comparison test.

Lung pathology was also examined in all hamsters, using a semiquantitative grading system to score the severity of the lung pathology (Fig. 7 and table S1). There was a significant difference in the lung pathology of control animals (*n* = 4) and PTX-COVID19-B–vaccinated animals (*n* = 4) after challenge with SARS-CoV-2. The main histopathologic feature after SARS-CoV-2 infection was extensive mixed inflammatory cell infiltration. The lung pathology was less extensive in the PTX-COVID19-B–vaccinated animals, although there was individual variability in grades. Together, these results indicate that vaccination with PTX-COVID19-B prevented productive infection of the lungs and upper respiratory tracts by SARS-CoV-2 in hamsters, and protected the animals from moderate/severe lung inflammation.

### Safety of PTX-COVID19-B in the animals

C57BL/6 mice and hamsters were checked daily during the experiments. The general status of the vaccinated animals such as appearance, feeding, and mobility was the same as of the control mice.

Male and female BALB/c mice immunized with 4- and 20-μg doses of PTX-COVID19-B were evaluated for body weight and injection site dermal scoring using a modified Draize scoring method (*41*) on days 23 and 43 (2 and 22 days after the second immunization, respectively). No differences in body weight were observed in immunized males compared to control mice. A small weight loss was observed in females compared to control animals in the week following the second injection of PTX-COVID19-B at the 20-μg dose, but by day 43, there was no significant difference in average body weight compared to control groups. A slight transient dermal erythema was observed in a small proportion (20%) of vaccinated mice, which disappeared by day 43. Therefore, PTX-COVID19-B intramuscular immunization in BALB/c mice was well tolerated, and only mild transient effects were observed, which resolved by day 43.

## DISCUSSION

The results presented here indicate that the SARS-CoV-2 mRNA vaccine PTX-COVID19-B is safe and effective in mouse and hamster models. Both rodent models have been widely used in assessing SARS-CoV-2 vaccines and therapeutics (*42*). Except for some VOCs and mouse-adapted strains that have N501Y mutation, SARS-CoV-2 S protein cannot bind mouse ACE2 efficiently to infect wild-type mice (*43*, *44*). Transgenic mice or mice transduced with viral vectors to express hACE2 are susceptible to SARS-CoV-2 infection, with high-level SARS-CoV-2 replication and ensuing inflammation in respiratory tracts. Due to the widespread expression of hACE2 in multiple tissues, transgenic mice exhibit pathology in multiple organs and usually succumb to death within a few days after infection (*45*). In contrast, mice transduced with AAV or adenovirus vectors express hACE2 primarily in lungs, where SARS-CoV-2 replication and ensuing pathology are restricted (*46*). Syrian hamsters are naturally susceptible to SARS-CoV-2 infection and display variable degrees of diseases including pneumonia. One remarkable feature of Syrian hamsters is that they can transmit SARS-CoV-2 infection, making them a critical model in studying SARS-CoV-2 transmission. Nonhuman primates (NHPs) are valuable models in studying SARS-CoV-2 because of their genetic and physiological resemblance to humans. However, SARS-CoV-2 infection of NHP usually only lead to self-limited mild disease (*47*). Availability and cost also limited their wide use. In the current study, we used mouse and hamster models since they allowed us to evaluate the safety, immunogenicity, and efficacy of PTX-COVID19-B in a fast and cost-effective way. Furthermore, the mouse model enabled us to do a thorough analysis of the immune responses elicited by PTX-COVID19-B. We found that PTX-COVID19-B elicited robust cellular and humoral immune responses and could completely protect vaccinated mice or hamsters from productive SARS-CoV-2 infection in the lungs. Although it is difficult to make a direct comparison because of variations in experimental conditions, the immunogenicity, such as the nominal titers of vaccine-induced nAb, and the efficacy of PTX-COVID19-B are comparable to those reported in the small animal studies of the other two mRNA vaccines approved for emergency use in humans (*19*, *48*).

PTX-COVID19-B also prevented SARS-CoV-2 replication in the upper respiratory tracts of hamsters, as shown by little or no infectious virus detected in nasal turbinates or oral swabs. Suppression of virus replication in upper respiratory tracts can reduce transmission of respiratory viruses but has been hard to achieve for respiratory virus vaccines, possibly because of the poor performance of these vaccines in inducing mucosal immunity in upper respiratory tracts (*49*, *50*). In this regard, some SARS-CoV-2 vaccines, including one of the two approved mRNA vaccines, were shown to be capable of suppressing the virus replication in both upper and lower respiratory tracts in animal studies (*19*, *48*, *51*, *52*). Additional experiments are needed to confirm the upper respiratory tract findings reported here, including the examination of the mucosal immune response elicited by PTX-COVID19-B and tests to see whether PTX-COVID19-B can prevent SARS-CoV-2 transmission between hamsters.

PTX-COVID19-B encodes a full-length membrane-anchored S protein derived from the ancestral Wuhan-Hu-1 isolate with a D614G substitution to match the predominant circulating SARS-CoV-2 strains at this amino acid location. During the preparation of this manuscript, SARS-CoV-2 VOCs emerged and have begun dominating the circulating strains worldwide, casting a doubt on the efficacy of current SARS-CoV-2 vaccines (*29*–*31*). Fortunately, immune sera from human subjects receiving the other two approved mRNA vaccines have been shown to neutralize many VOCs, although usually with reduced efficacy, in particular for the Beta lineage (*6*–*9*, *53*). Consistent with these reports, PTX-COVID19-B–elicited mouse immune sera were still capable of neutralizing four dominant VOCs with high potency by a pseudovirus assay. However, further experiments are needed to test the efficacy of PTX-COVID19-B in protection of animals against infections by VOCs.

The 2P mutation (K986P and V987P) was reported to stabilize the ectodomain of the S protein in the prefusion conformation (*15*), which was regarded as crucial in inducing nAb, and thus was adopted in some SARS-CoV-2 vaccines, including the currently approved Pfizer’s and Moderna’s mRNA vaccines (*19*, *48*, *54*, *55*). PTX-COVID19-B does not have the 2P mutation since we thought it likely that the membrane anchor could stabilize the full-length S in the prefusion conformation, as reported for other virus envelope proteins (*56*–*58*). Furthermore, the structure of S protein with the 2P mutation (S-2P) was shown to be different from that of native S protein, assuming a more open and more RBD-up conformation, which may affect the induction of nAb (*59*). In this regard, multiple studies showed that antibody response elicited by Pfizer’s or Moderna’s mRNA vaccine was different from that stimulated by SARS-CoV-2 infection. For example, Amanat *et al.* (*60*) found that Pfizer’s mRNA vaccine tilted toward inducing non-nAbs compared to SARS-CoV-2 infection. More recently, Chen *et al.* (*61*) reported that although Pfizer’s or Moderna’s mRNA vaccine initially elicited higher antibodies than SARS-CoV-2 infection, S- and RBD-binding antibodies induced by SARS-CoV-2 infection were more durable. Besides, nAbs induced by SARS-CoV-2 infection were broader, i.e., more capable of neutralizing VOCs, than those elicited by either Pfizer’s or Moderna’s mRNA vaccine (*61*). The mechanisms underlying the differences in antibody response between the two mRNA vaccines and SARS-CoV-2 infection are not known yet, but collectively, these findings suggest that native S protein may be superior to S-2P in inducing long-term immunity. G614 in the S protein encoded by PTX-COVID19-B may additionally enhance the stability of the expressed S trimer in prefusion conformation, as shown by a recent structural study (*62*), which can help induce better nAb as well. Notably, S protein in Pfizer’s and Moderna’s currently authorized mRNA vaccines has D614 in addition to the 2P mutation. In the current study, we found that PTX-COVID19-B elicited equivalent levels of SARS-CoV-2 nAb and afforded similar protection from SARS-CoV-2 infection as compared to Pfizer’s and Moderna’s vaccines within a few weeks after vaccination (*19*, *48*, *62*). We will follow up vaccinated animals and human subjects for long term to determine whether PTX-COVID19-B will elicit more durable nAb and provide more sustainable protection from SARS-CoV-2 infection than the currently authorized mRNA vaccines. An additional example for the use of wild-type full-length S in a SARS-CoV-2 vaccine is ChAdOx1 nCoV-19 (AZD1222), which was reported assuming the prefusion conformation on the cell surface (*63*). We also designed and tested another S construct, S_furinmut_, in which the furin cleavage site between S1 and S2 subunits was removed. Removing this site was presumed to stabilize the ectodomain of the S protein by keeping the S1 subunit from shedding from S2 and was used in some SARS-CoV-2 vaccines (*15*, *54*, *55*). However, we did not find that the S_furinmut_ mRNA performed better in eliciting nAb in mice compared to the S mRNA. G614 in the S protein of PTX-COVID19-B may obviate the need of furin cleavage site mutation to keep S1 linked to S2, as suggested by the structural study mentioned above (*62*). Additional experiments are required to test whether other modifications of the S protein could enhance the strength and breadth of the immunogenicity and efficacy of PTX-COVID19-B. In contrast to the S mRNA and S_furinmut_ mRNA, the RBD mRNA performed poorly in inducing a nAb response. This is consistent with previous reports showing that RBD was weakly immunogenic, possibly because of its small size (*64*, *65*). In addition, full-length S can elicit nAb targeting epitopes beyond RBD, such as in the N-terminal domain (NTD) and S2 subunit, and these non–RBD-targeting nAbs can work additively or synergistically with the RBD-targeting nAb to neutralize SARS-CoV-2 (*66*).

The protection mechanisms of SARS-CoV-2 vaccines have not been fully elucidated in humans, although nAb and T cells are assumed to be critical protection correlates (*36*, *49*, *67*). This is supported by animal studies where nAb responses were pivotal in protecting monkeys from SARS-CoV-2 infection, with CD8^+^ T cells also participating in protection (*68*). In addition, CD4^+^ T cell help is vital for the quantity and quality of nAb and CD8^+^ T cell responses against virus infection (*69*–*71*). T cells could also continue to attack the VOCs that escape the nAb response, since they recognize multiple linear epitopes, including epitopes in the conserved region of the S protein (*72*). In this regard, PTX-COVID19-B elicited both robust nAb and CD8^+^ T cell responses. PTX-COVID19-B also induced a predominant T_H_1 response, which is regarded as a desirable feature for respiratory virus vaccines (*73*). Additional experiments will be required to track nAb and T cell responses induced by PTX-COVID19-B, including their durability and capability to protect against VOCs.

The epitopes in the S protein targeted by nAb elicited by SARS-CoV-2 vaccines are not completely defined yet. Recent studies showed that, like the nAb generated from convalescent subjects, nAb from SARS-CoV-2 vaccinees mainly targets RBD, but NTD and, possibly, S2 also account for some of the neutralizing epitopes (*8*, *60*, *74*). Although we have not performed epitope mapping of the nAb elicited by PTX-COVID19-B, the VOCs’ neutralization data presented here suggest that PTX-COVID19-B induced polyclonal nAb targeting multiple epitopes, especially epitopes in RBD. For example, the marked reduction of neutralization potency of the immune sera against VOC Beta (B.1.351) suggests that the main epitopes comprise K417, E484, and/or N501 in RBD, which are mutated to N/K/Y in this VOC. Schmidt *et al.* (*75*) recently reported that an mRNA vaccine elicited much broader polyclonal nAb in subjects who were previously infected by SARS-CoV-2 than subjects who were not, suggesting that, in convalescent subjects, the mRNA vaccine induced nAb targeting more conserved epitopes. Fine epitope mapping of the nAb from vaccinees, including convalescent subjects receiving vaccination, may help design next-generation broad-spectrum SARS-CoV-2 mRNA vaccines.

We used both a live virus and pseudoviruses to measure neutralization of SARS-CoV-2 by the immune sera from the vaccinated animals. Neutralization of virus by antibody is usually defined as the capacity of antibody per se in reducing viral infectivity without the involvement of effector cells or other molecules (*76*). For SARS-CoV-2, binding of nAbs to the S protein blocks viral infection by impeding the interaction between S protein and ACE2. Other mechanisms, such as triggering premature transition of S protein to postfusion conformation, are also responsible for the neutralization (*77*). As shown here and by others, both live virus assay and pseudovirus assay are rigorous in quantifying neutralization of SARS-CoV-2, and the results from both assays correlate well with each other (*78*).

Although a few vaccines have been approved for emergency use in humans, additional safe, effective, and easily deployable SARS-CoV-2 vaccines are needed to meet the enormous challenge for the global immunization required to end the COVID-19 pandemic. On the basis of the results reported here, PTX-COVID19-B has been authorized by Health Canada in December 2020 to enter a phase 1 clinical trial (ClinicalTrials.gov number NCT04765436). Interim results of the phase 1 clinical trial showed excellent immunogenicity and safety/tolerability results. A phase 2 clinical trial is ongoing in Canada, with a phase 3 clinical trial at planning stage. Results of these clinical trials will determine whether PTX-COVID19-B will eventually be added into the vaccine arsenal in the global fight against SARS-CoV-2.

## MATERIALS AND METHODS

### Study design

The objective of this study was to evaluate the immunogenicity, safety, and efficacy of a SARS-CoV-2 mRNA vaccine in mice and hamsters. The sample size of mice was determined by power analysis assuming 60% protection efficacy. Because of the capacity limit in our animal facility, a total of 16 hamsters in two groups were used in this study. All animals were randomly assigned to different treatment groups. The performers measuring SARS-CoV-2 neutralization by mouse sera and SARS-CoV-2 virus in mouse tissues and the pathologist examining animal pathology were blinded to the sample groupings.

### Ethics

All animal work was approved by the Animal Care Committees of the University of Toronto. For studies involving human samples, written informed consent was obtained from convalescent COVID-19 patients, and samples were obtained and used according to a research ethics board (REB)–approved protocol (St. Michael’s Hospital REB20-044c to M.A.O.).

### Vaccine

The mRNAs used in these studies encode the full-length S (amino acids 1 to 1273), the S_furinmut_ that is the same as the full-length S except for the furin cleavage site NSPRRA (amino acids 679 to 684) changed to IL, or the RBD (amino acids 319 to 541). The amino acid sequences encoded by these mRNAs are the same as the Spike protein sequence from SARS-CoV-2 Wuhan-Hu-1 isolate, GenBank accession number MN908947.3, except for a D614G substitution in S mRNA and S_furinmut_ mRNA. The mRNAs contain codon-optimized open reading frames for the S, S_furinmut_, or RBD flanked by an optimized capped 5′ untranslated region (5′UTR) and an optimized 3′UTR followed by a polyadenylated tail. Codon and sequence optimization were performed by using Providence’s proprietary algorithm. The mRNA was produced by in vitro transcription of a linear plasmid template using T7 RNA polymerase. The mRNA was purified by Providence’s proprietary purification process using a series of purification steps to remove transcription enzymes, the linear DNA template, and mRNA-related impurities before formulation. LNPs were prepared by mixing a buffered solution of mRNA with an ethanol solution of lipids [distearoylphosphatidylcholine (DSPC), cholesterol, polyethylene glycol–lipid, and ionizable lipid] following Genevant’s proprietary process (Vancouver, BC, Canada). The LNPs were concentrated by tangential flow ultrafiltration and then diafiltered against an aqueous buffer system. Following a 0.2-μm filtration process, the LNPs were subjected to quality tests including RNA concentration, encapsulation efficiency, particle size, pH, and osmolality.

### mRNA transfection of HEK293T cells and detection of the expressed immunogens

S mRNA, S_furinmut_ mRNA, and RBD mRNA were transfected into HEK293T cells using Lipofectamine MessengerMAX transfection reagent (Thermo Fisher Scientific, Mississauga, ON, Canada) according to the manufacturer’s protocol. Briefly, HEK293T cells cultured in Dulbecco’s modified Eagle’s medium (DMEM)–10 medium [DMEM supplemented with 10% fetal bovine serum (FBS), 100 U of penicillin, 100 μg of streptomycin, and 2 mM l-glutamine; DMEM was purchased from Thermo Fisher Scientific, and all others were purchased from Wisent Bioproducts, St-Bruno, QC, Canada] were seeded into six-well plates (Corning Life Sciences, Tewksbury, MA). After overnight culture, 2 μg of mRNAs was diluted in 125 μl of Opti-MEM (Thermo Fisher Scientific), mixed with the Lipofectamine MessengerMAX transfection reagent, and then added to the cells. Twenty-four hours later, the supernatant was collected from the transfected cells for an in-house sandwich ELISA to detect the expressed immunogens in the supernatant. Cells were collected for the detection of the expressed immunogens on the cell surface by flow cytometry.

For the sandwich ELISA, Immulon 2HB flat-bottom microtiter plates (Thermo Fisher Scientific) were coated with an RBD-specific neutralizing mAb COV2-2165 (provided by J. E. Crowe Jr. from Vanderbilt University Medical Center, Nashville, TN), washed, and blocked with DPBS containing 3% bovine serum albumin (Sigma-Aldrich, Oakville, ON, Canada). The supernatant was then pipetted into the plates. Purified RBD protein (provided by J. M. Rini, Department of Biochemistry, University of Toronto, Toronto, ON, Canada) was also added to the plates as a positive control. After 2 hours of incubation, the plates were washed and mouse anti-S immune serum (provided by J. R. Carlyle from the Department of Immunology, University of Toronto, Toronto, ON, Canada) was added to the plates. After 1 hour of incubation, the plates were washed and horseradish peroxidase (HRP)–labeled goat anti-mouse IgG (SouthernBiotech, Birmingham, AL) was added. After 1 hour of incubation, SureBlue 3,3′, 5, 5′ - tetramethylbenzidine (TMB) microwell peroxidase substrate (KPL, Gaithersburg, MD) was added, and 15 min later, 1 N HCl was pipetted into the plates to stop the reaction. OD_450_ (optical density at 450 nm) was then read using a microplate reader (Thermo Fisher Scientific).

For flow cytometry, cells were first stained with the RBD-specific neutralizing mAb COV2-2165 and then with an allophycocyanin mouse anti-human IgG (BD Biosciences, Mississauga, ON, Canada). Stained cells were run on LSRFortessa (BD Biosciences). FlowJo (BD Biosciences) was used to analyze the flow cytometry data.

### Mouse vaccination

Female C57BL/6 mice of 6 to 8 weeks old were vaccinated intramuscularly twice with a 3-week interval. In some experiments, both male and female BALB/c mice of 6 to 8 weeks old were used. Various doses of mRNA vaccines or control tdTomato mRNA in 50-μl total volume were injected into the hind leg muscle for each immunization. Naïve mice received the same volume of either DPBS or the vaccine formulation buffer. Each day before vaccination, blood was collected from the mice through the saphenous vein. Three weeks after boost vaccination, mice were humanely euthanized, and spleen and blood samples were collected. Serum was isolated from the blood by centrifugation at 10,000*g* for 30 s at 4°C.

### S-specific immunoglobulin ELISA

ELISAs were performed as previously described with minor modifications for mouse samples (*79*). Ninety-six–well plates (Green BioResearch, Baton Rouge, LA) were coated with 200 ng per well of recombinant purified full-length spike trimer SmT1 (*80*) and blocked with 3% (w/v) milk powder in PBS–Tween 20 (PBS-T). Serial dilutions of mouse samples in 1% (w/v) milk powder in PBS-T were added to the plate (starting at 1:100 dilution with fivefold dilutions) and incubated for 2 hours at room temperature. Wells were then washed three times with 200 μl of PBS-T before incubation for 1 hour with secondary antibodies (HRP-labeled goat anti-mouse IgG1/IgG2b/IgG2c purchased from SouthernBiotech or HRP-labeled goat anti-mouse IgG Fcγ purchased from Jackson ImmunoResearch, West Grove, PA) in 1% (w/v) milk powder in PBS-T. Samples were washed three times with PBS-T, and then 1-Step Ultra TMB-ELISA Substrate Solution (Thermo Fisher Scientific) was added for 15 min at room temperature. The reaction was quenched with an equal volume of stop solution containing 0.16 N sulfuric acid (Thermo Fisher Scientific). Plates were read using a spectrophotometer (BioTek Instruments Inc., Cytation 3) reading at 450 nm. All sample raw OD values had blank values subtracted before analysis. OD values of each PTX-COVID19-B–vaccinated mouse serum minus average of OD values of four tdTomato control mouse sera at the same dilution were used to calculate the EC_50_ titer using the four-parameter logistic regression analysis in GraphPad Prism 8 (GraphPad Software, La Jolla, CA).

### Serum neutralization using SARS-CoV-2 virus

A microneutralization assay was used to measure the neutralizing titers of the sera (*34*). Briefly, VeroE6 cells cultured in DMEM-10 were seeded into 96-well plates and cultured overnight. Sera were heat-inactivated at 56°C for 30 min. Serial dilutions of the sera were mixed with 100 TCID_50_ SARS-CoV-2 virus [isolate SARS-CoV-2-SB2-P3 PB clone 1, passage 3 (*28*)] in serum-free DMEM, incubated at 37°C for 1 hour, and then added onto the VeroE6 cells. The cell plates were then incubated at 37°C for 1 hour, shaking every 15 min. Inoculums were then removed, and DMEM-2 (DMEM supplemented with 2% FBS, 100 U of penicillin, 100 μg of streptomycin, and 2 mM l-glutamine) was added to the cells. Cell plates were incubated at 37°C for 5 days, and cytopathic effect (CPE) was checked every day. ID_50_ titer was defined as the highest dilution factor of the serum that protected 50% of the cells from CPE and calculated by using the four-parameter logistic regression analysis in GraphPad Prism 8. The performer of the assay was blinded to the grouping of the mice.

### Serum neutralization using pseudovirus

Spike-pseudotyped lentiviral assays were performed as previously described with reagents provided by J. D. Bloom (Department of Genome Sciences, University of Washington, Seattle, WA) and with minor modifications for mouse samples (*79*). Briefly, Spike-pseudotyped lentivirus particles (both wild-type Wuhan-Hu-1 and tested VOCs) were generated and used at ~1:25 virus stock dilution (a virus dilution resulting in >1000 relative luciferase units over control). For the neutralization assay, diluted mouse sera (1:40 from stock sera) were serially diluted (from 2.5- to 4-fold dilutions over seven dilutions to encompass a complete neutralization curve per sample) and incubated with diluted pseudovirus at a 1:1 ratio for 1 hour at 37°C before being transferred to plated HEK293T-ACE2/TMPRSS2 cells and incubated for an additional 48 hours at 37°C and 5% CO_2_. After 48 hours, cells were lysed, and Bright-Glo luciferase reagent (Promega, Madison, WI) was added for 2 min before reading with a PerkinElmer Envision instrument (PerkinElmer, Waltham, MA). ID_50_ titers were calculated with nonlinear regression (log[inhibitor] versus normalized response − variable slope) using GraphPad Prism 8. The assay was performed in the same manner for all VOCs tested. The performer of the assay was blinded to the grouping of the mice.

### ELISPOT assay

To perform IFN-γ ELISPOT for C57BL/6 mice, ELISPOT plates (Sigma-Aldrich) were coated with rat anti-mouse IFN-γ antibody (BD Biosciences) overnight. Plates were washed and blocked with RPMI-10 medium (RPMI 1640 supplemented with 10% FBS, 100 U of penicillin, 100 μg of streptomycin, and 2 mM l-glutamine; all were purchased from Wisent Bioproducts) for 2 hours. Splenocytes were added into the plates and stimulated with a SARS-CoV-2 S peptide pool (15-mer peptides with 11–amino acid overlap covering the full-length S, total 315 peptides; JPT Peptide Technologies GmbH, Berlin, Germany) at 1 μg/ml per peptide. The same volume of 40% dimethyl sulfoxide (DMSO; Sigma-Aldrich), the solution to dissolve the peptide pool, was used as the negative control. Phorbol 12-myristate 13-acetate (PMA)/ionomycin (Sigma-Aldrich) was used as the positive control. After overnight incubation, the cells were washed away, biotinylated anti-mouse IFN-γ (BD Biosciences) was added, and the plates were incubated for 2 hours. After washing with PBS/0.01% Tween 20, streptavidin-HRP enzyme conjugate (Thermo Fisher Scientific) was added into the plates, which was incubated for 1 hour. After washing with PBS/0.01% Tween 20, TMB ELISPOT substrate (Mabtech, Cincinnati, OH) was added into the plates, and the spots were developed and read with an ImmunoSpot analyzer (Cellular Technology Limited, Cleveland, OH). The number of the S-specific spots was acquired by subtracting the number of the spots of the DMSO control wells from the number of the spots of the corresponding S peptide pool stimulation wells.

For IFN-γ and IL-4 ELISPOT of BALB/c mice, similar procedures as described above were performed using an ImmunoSpot mouse IFN-γ and IL-4 ELISPOT kit (CTL, Shaker Heights, OH), with modifications according to the manufacturer’s protocol. Splenocytes were stimulated with two subpools (158 and 157 peptides, respectively) of JPT’s SARS-CoV-2 S peptide pool separately. The number of the S-specific spots was acquired by adding up the numbers of the spots in each subpool-stimulated splenocyte population minus the number of the spots of the DMSO control wells.

### T cell intracellular cytokine staining

Mouse splenocytes were cultured in RPMI-10 and stimulated with the S peptide pool at 1 μg/ml per peptide in the presence of GolgiStop and GolgiPlug (BD Biosciences) for 6 hours. DMSO (40%) and PMA/ionomycin were used as the negative and positive control, respectively. Cells were first stained with the LIVE/DEAD Fixable Violet Dead Cell Stain, blocked the FcR with TruStain FcX (BioLegend, San Diego, CA), and then stained with the fluorochrome-labeled anti-mouse CD3/CD4/CD8/CD44/CD62L mAbs (all purchased from BioLegend, except CD44 from BD Biosciences). Cells were then treated with Cytofix/Cytoperm (BD Biosciences) and stained with fluorochrome-labeled anti-mouse IFN-γ/TNF-α/IL-2/IL-4/IL-5 mAbs (BioLegend). LSRFortessa was used to acquire the flow cytometry data, which were then analyzed with FlowJo. Percentage of cytokine^+^ T cells was calculated by subtracting the percentage of the DMSO control cells from the percentage of the corresponding S peptide pool stimulation cells.

### Multiplex immunoassay

Supernatant from the mouse splenocytes stimulated with the S peptide pool at 1 μg/ml per peptide for 24 hours was collected, and cytokines were detected using a multiplex capture sandwich immunoassay. A Bio-Plex Pro mouse cytokine T_H_1/T_H_2 assay kit (Bio-Rad, Mississauga, ON, Canada) was used. Both standards and samples were prepared following the manufacturer’s instructions. The assay plate was read in a Bio-Plex MAGPIX system (Bio-Rad), and data were analyzed using the Bio-Plex Manager Software (Bio-Rad).

### SARS-CoV-2 mouse challenge

Mice were anesthetized with isoflurane and intranasally transduced with 10^11^ genomic copies of the AAV6-hACE2 or, in some experiments, AAV6-luciferase as control (provided by S. K. Wootton, Department of Pathobiology, University of Guelph, Guelph, ON, Canada). Nine days later, the mice were anesthetized with isoflurane and intranasally challenged with 10^5^ TCID_50_ SARS-CoV-2 (SARS-CoV-2, isolate Canada/ON/VIDO-01/2020, GISAID (Global Initiative on Sharing Avian Influenza Data) accession number EPI_ISL_425177). At 4 dpi, mice were humanely euthanized, and blood and lungs were collected. For each mouse, the left lung was sent for pathology examination, and the right lung was homogenized in DMEM-2, using a bead mill homogenizer (OMNI International, Kennesaw, GA). Lung homogenates were then clarified by centrifugation at 10,000 rpm for 5 min.

### Hamster vaccination and SARS-CoV-2 challenge

Male Syrian hamsters, aged 6 to 10 weeks, were obtained from Charles River Canada (Saint-Constant, QC, Canada). The animals were kept in Biosafety Level-2 (BSL-2) housing until virus challenge in Biosafety Level-3 (BSL-3) in vivo facility. A total of two groups of 16 animals (*n* = 8 per group) were immunized twice with a 3-week interval with 20 μg of PTX-COVID19-B in 100 μl via intramuscular route into the rear limbs (50 μl per limb). Mock animal group received an equivalent volume of PBS. Three weeks after boost, animals were intranasally challenged with SARS-CoV-2 (1 × 10^4^ TCID_50_ in 100 μl per animal) under inhaled isoflurane anesthesia. Animals were monitored daily for clinical signs of disease, and phenotype parameters such as weight loss and body temperature were recorded every second day. No death was recorded after the viral infection.

Four animals in both challenge groups were humanely euthanized at 4 and 8 dpi for virological and histopathological analyses. Blood and major organ tissues were collected, and the tissues were separated into two parts: one part immediately fixed in 10% formalin, and the other part immediately frozen at −80°C until further use. The frozen tissue samples were homogenized in 1 ml of DMEM-2 manually in a disposable 15-ml closed tissue grinder system (Thermo Fisher Scientific). A total of 140 μl out of 1-ml samples was used for RNA extraction, while 500 μl of homogenates was used for quantification of SARS-CoV-2. For oral swabs, anesthetized animals were swabbed (9- to 11-s swabbing), and swabs were then introduced into 1 ml of DMEM-2. All oral swab samples were frozen until further processing. A total of 500 μl of each oral swab sample was used for quantification of SARS-CoV-2.

### Determination of infectious SARS-CoV-2 titer

VeroE6 cells cultured in DMEM-10 were seeded into 96-well plates and incubated overnight at 37°C. On the following day, culture medium was removed and tissue samples 10-fold serially diluted in DMEM supplemented with 1% FBS were added onto the cells. The plates were then incubated at 37°C for 1 hour. After incubation, lung homogenates were replaced with 100 μl per well of DMEM-2, and the cells were incubated at 37°C for 5 days. CPE was checked on days 3 and 5. TCID_50_ was defined as the highest dilution factor of the inoculum that yielded 50% of the cells with CPE and determined by using the Spearman-Karber TCID_50_ method.

### Real-time RT-PCR

Real-time RT-PCR to quantify the genomic copies of SARS-CoV-2 in tissue homogenates was performed according to the published protocol (*28*). Briefly, RNA was isolated from the tissue homogenates using a QIAamp Viral RNA kit (QIAGEN, Toronto, ON, Canada). A Luna Universal Probe One-step RT-qPCR kit (New England Biolabs, Ipswich, MA) was used to amplify the envelope (E) gene using the following primers and probes: ACAGGTACGTTAATAGTTAATAGCGT (forward) and ATATTGCAGCAGTACGCACACA (reverse) and probe CAL Fluor Orange 560-ACACTAGCCATCCTTACTGCGCTTCG-BHQ-1. The cycling conditions were 1 cycle at 60°C for 10 min, then 95°C for 2 min, followed by 44 cycles at 95°C for 10 s and 60°C for 15 s. An E gene DNA standard (pUC57-2019-nCoV-PC:E, GenScript, Piscataway, NJ) was also run at the same time for conversion of *C*_t_ value to genomic copies by using the Rotor-Gene Q software (QIAGEN).

### Pathology

The formalin-fixed lung tissue was processed for paraffin embedding and microtomy and then stained with hematoxylin and eosin. The blocks were examined at three separate levels (three separate slides). Histological sections were examined blind to vaccination status. Semiquantitative grading of lung was conducted according to table S1.

### Statistical analysis

One-way analysis of variance (ANOVA) (Kruskal-Wallis test) followed by Dunn’s multiple comparison, two-way ANOVA followed by Sidak’s multiple comparison, two-tailed paired *t* test, or two-tailed unpaired *t* test (Mann-Whitney) was used for comparison between groups, as indicated in the figure legends. Spearman correlation test was used for correlation analysis. Logistic regression was used for determining nAb ID_50_ threshold titer that would confer 95% predicted probability of protection from productive SARS-CoV-2 infection in mice. All statistical analysis was performed by using GraphPad Prism 8. *P* < 0.05 was regarded as statistically significant.

## Notes

View/request a protocol for this paper from *Bio-protocol*.
